# The parasubthalamic nucleus refeeding ensemble delays feeding initiation and hastens water drinking

**DOI:** 10.1038/s41380-024-02653-y

**Published:** 2024-07-04

**Authors:** Jeffery L. Dunning, Catherine Lopez, Colton Krull, Max Kreifeldt, Maggie Angelo, Leeann Shu, Charu Ramakrishnan, Karl Deisseroth, Candice Contet

**Affiliations:** 1 Department of Molecular Medicine, The Scripps Research Institute, La Jolla, CA USA; 2 Department of Bioengineering, Stanford University, Stanford, CA USA; 3 Howard Hughes Medical Institute, Stanford University, Stanford, CA USA; 4 Department of Psychiatry and Behavioral Sciences, Stanford University, Stanford, CA USA

**Keywords:** Neuroscience, Physiology

## Abstract

The parasubthalamic nucleus (PSTN) is activated by refeeding after food deprivation and several PSTN subpopulations have been shown to suppress feeding. However, no study to date directly addressed the role of PSTN neurons activated upon food access in the control of ensuing food consumption. Here we identify consumption latency as a sensitive behavioral indicator of PSTN activity, and show that, in hungry mice, the ensemble of refeeding-activated PSTN neurons drastically increases the latency to initiate refeeding with both familiar and a novel, familiar food, but does not control the amount of food consumed. In thirsty mice, this ensemble also delays sucrose consumption but accelerates water consumption, possibly reflecting anticipatory prandial thirst, with again no influence on the amount of fluid consumed. We next sought to identify which subpopulations of PSTN neurons might be driving these latency effects, using cell-type and pathway-specific chemogenetic manipulations. Our results suggest a prominent role of PSTN *Tac1* neurons projecting to the central amygdala in the hindrance of feeding initiation. While PSTN *Crh* neurons also delay the latency of hungry mice to ingest familiar foods, they surprisingly promote the consumption of novel, palatable substances. Furthermore, PSTN *Crh* neurons projecting to the bed nucleus of the stria terminalis accelerate rehydration in thirsty mice. Our results demonstrate the key role of endogenous PSTN activity in the control of feeding and drinking initiation and delineate specific circuits mediating these effects, which may have relevance for eating disorders.

## Introduction

The hypothalamus is a critical hub for the integration of interoceptive signals and the initiation of homeostatic behaviors regulating need states such as hunger and thirst. In particular, molecularly defined subpopulations of neurons in the arcuate nucleus, paraventricular nucleus, and lateral hypothalamic area control food intake [1, 2], while median preoptic nucleus excitatory and inhibitory neurons promote and suppress, respectively, water drinking [3, 4]. Beyond physiological needs, hypothalamic nuclei also control food and fluid consumption driven by hedonic motives (i.e., pleasurable sensory perception or affective state), as they interface with corticolimbic and midbrain monoaminergic systems that encode reward and decision making [5, 6].

In recent years, the parasubthalamic nucleus (PSTN), a differentiation of the premammillary lateral hypothalamic area that is highly interconnected with brain regions regulating interoception and appetite, has emerged as a new player in the regulation of ingestive behaviors (see [7] for review). Significant induction of c-Fos expression, a marker of neuronal activation, has been observed in the rodent PSTN following predatory hunting of cockroaches, early active phase surge of food intake, refeeding after food deprivation, anorexia-inducing amino-acid deficient diet refeeding, and sucrose drinking following water deprivation [8–14]. While all PSTN neurons are glutamatergic (VGluT2-positive), they comprise subpopulations expressing high levels of either *Tac1* (encoding preprotackykinin-A, a precursor of substance P and neurokinin A) or *Crh* (encoding corticotropin-releasing factor, CRF) with minimal overlap [14–16]. Both PSTN^*Tac1*^ and PSTN^*Crh*^ neurons show strong activation in response to refeeding in food-deprived mice [14].

Manipulations of PSTN neurons via optogenetic, chemogenetic, and targeted cell ablation approaches have further demonstrated their functional implication in consummatory behaviors (see [7] for review). Specifically, stimulation of PSTN^VGluT2^ neurons projecting to the paraventricular nucleus of the thalamus (PVT), PSTN^*Tac1*^ neurons as a whole, as well as PSTN^*Tac1*^ neurons projecting to the central nucleus of the amygdala (CeA), PVT, parabrachial nucleus, or nucleus of the tractus solitarius, reduces food intake in ad libitum fed mice [14, 17]. This effect is not observed when stimulating PSTN^*Crh*^ neurons or PSTN^*Tac1*^ neurons projecting to the bed nucleus of the stria terminalis (BNST), highlighting the cell-type and pathway specificity of the PSTN’s influence on food intake [14]. Activation of PSTN^*Adcyap1*^ neurons, which overlap with both the *Tac1* and *Crh* populations, also reduces the time spent feeding in hungry mice regaining access to food [18]. On the other hand, inhibiting PSTN^*Tac1*^ somas counters the anorectic effect of a malaise-inducing agent (lipopolysaccharide), neophobia (first-time exposure to sucrose), or appetite-suppressing hormones (amylin, cholecystokinin, peptide YY), while ablating PSTN^*VGluT2*^ neurons abolishes anorexia induced by glucagon-like peptide-1 [12, 14, 19] and silencing PSTN neurons as a whole (CaMKIIα promoter) attenuates cholecystokinin-induced and fear-induced feeding suppression [18, 20]. While these studies elegantly demonstrated the ability of several PSTN subpopulations to suppress feeding, none of them directly addressed the role of PSTN neurons activated upon food access resumption in hungry animals in the control of ensuing food consumption. To address this gap of knowledge, we used chemogenetics in “Targeted Recombination in Active Populations” mice (TRAP2 mice, in which the sequence encoding iCre-ER^T2^ is inserted in the *Fos* locus without disrupting endogenous *Fos* expression [21]), to selectively re-activate the ensemble of refeeding-activated PSTN neurons and determine their influence on consummatory behaviors. Moreover, in addition to measuring the amount of food and fluid consumed (as was done in previous studies), we recorded the latency to first bite of food or first lick of fluid as a behavioral indicator of the mouse’s motivation to eat or drink, which emerged as highly sensitive to PSTN manipulations.

We found that, in hungry mice, the PSTN refeeding ensemble drastically increases the latency to initiate refeeding with both familiar chow and a novel, palatable food (Froot Loops), but does not control the amount of food consumed. In thirsty mice, this ensemble also delays sucrose consumption but accelerates water consumption, with again no influence on the amount of fluid consumed. Given that previous studies only reported measures of intake (i.e., amount consumed), we next sought to examine which subpopulations of PSTN neurons might be driving these latency effects, using cell-type and pathway-specific chemogenetic manipulations.

## Materials and methods

### Animals

TRAP2 (Fos^tm2.1(icre/ERT2)Luo/J^, stock #030323 [21]), *Crh*-Cre (*Crh*-IRES-Cre, B6(Cg)-Crh^tm1(cre)Zjh/J^, stock #012704 [22]), and *Tac1*-Cre (B6;129S-Tac1^tm1.1(cre)Hze/J^, stock #021877 [23]), breeders were obtained from The Jackson Laboratory. C57BL/6 J mice were obtained from Scripps Research rodent breeding colony. All mice used for experimentation were heterozygous for the Cre allele. All experimental subgroups contained a mix of age-matched males and females. Mice from the same litter were distributed across experimental subgroups.

Mice were maintained on a 12/12 h light/dark cycle. Food (Teklad LM-485, Envigo) and reverse osmosis purified water were available ad libitum, except for a 24-h period of food deprivation or 4-h water deprivation in relevant experiments. All mice were single-housed in static caging with Sani-Chips (Envigo) bedding one week prior to behavioral assays and remained in these housing conditions for the duration of experimentation. All mice were at least 10 weeks old at the time of surgery. All procedures adhered to the National Institutes of Health Guide for the Care and Use of Laboratory Animals and were approved by the Institutional Animal Care and Use Committee of The Scripps Research Institute.

### Drugs

For iCre-ER^T2^ activation in TRAP2 mice [21, 24], the estrogen receptor ligand 4-hydroxytamoxifen (4-OHT) was obtained from Hello Bio (HB6040). 4-OHT was dissolved in 100% ethanol and then mixed 1:1 with a mixture of 1 part castor oil (Sigma-Aldrich 259853) and 4 parts sunflower oil (Sigma-Aldrich S5007). Ethanol was then removed via vacuum centrifugation and the remaining oil was again diluted in the same 1:4 oil mixture to achieve a concentration of 10 mg/mL before intraperitoneal (i.p.) injection at a dose of 50 mg/kg (5 mL/kg injection volume, 23-gauge needle). Clozapine-N-oxide (CNO) was used as the ligand for hM3Dq and hM4Di designer receptors [25, 26] and was obtained from Enzo Life Sciences Inc. (freebase, BML-NS105-0025) or Hello Bio (dihydrochloride, HB6149). CNO freebase was dissolved in dimethyl sulfoxide (DMSO) and diluted in 0.9% saline (0.5% final DMSO concentration) and CNO dihydrochloride was dissolved directly in saline for i.p. injection (3 mg/kg body weight, 10 mL/kg injection volume, 27-gauge needle). Salvinorin B (SalB) was used as the ligand for the κ-opioid receptor-based designer receptor (KORD [27]) and was obtained from Hello Bio (HB4887). SalB was dissolved in 100% DMSO for subcutaneous (s.c.) injection (10 mg/kg body weight, 1 mL/kg injection volume using a Hamilton 250 μL syringe #81108, 27 gauge, point style 4 needle). Prior to SalB testing, mice were habituated for two days to DMSO s.c. injections.

### Viral vectors

Adeno-associated viral serotype 2 (AAV2) vectors encoding the hM3Dq excitatory or hM4Di inhibitory designer receptor fused to the red fluorescent protein mCherry, or mCherry alone, under the control of the human synapsin promoter (hSyn) and in a Cre-dependent manner (Double-floxed Inverted Open reading frame, DIO), were obtained from the Vector Core at the University of North Carolina at Chapel Hill (AAV2-hSyn-DIO-hM3Dq-mCherry, Addgene plasmid # 44361, lot 8269, titer 1.5 × 10^13^ vg/mL; AAV2-hSyn-DIO-hM4Di-mCherry, Addgene plasmid # 44362, lot 8268, titer 1.8 × 10^13^ vg/mL; AAV2-hSyn-DIO-mCherry, Addgene plasmid # 50459, lot 8267, titer 1.3 × 10^13^ vg/mL) [26]. An AAV8 encoding the inhibitory designer receptor KORD fused to the fluorescent protein mCitrine under the control of the human synapsin promoter in a Cre-dependent manner was obtained from Addgene (AAV8-hSyn-dF-HA-KORD-IRES-mCitrine, viral prep # 65417-AAV8, lot v43122, titer 2.1 × 10^13^ gc/mL) [27]. A retrograde AAV [28] encoding the Cre enzyme fused to the green fluorescent protein (GFP) under the control of the human synapsin promoter was obtained from Addgene (AAVrg.hSyn.HI.eGFP-Cre.WPRE.SV40, viral prep # 105540-AAVrg, lot V102961, titer 2.5 × 10^13^ gc/mL). An AAV8 vector expressing hM3Dq-mCherry under a short EF1α promoter in a Cre- and Flp- dependent manner was generated, packaged, and purified by the laboratory of Karl Deisseroth (AAV8-nEF-Con/Fon-hM3Dq-mCherry, lot 7280, titer 1.23 × 10^12^ gc/mL) and used in conjunction with a retrograde AAV encoding the Flpo enzyme under an EF1α promoter (AAVrg-EF1a-Flpo, Addgene viral prep # 55637-AAVrg, lot v56725, titer 1.02 × 10^12^ gc/mL) [29].

### Experimental cohorts

The data were collected from seven separate cohorts of mice.

A cohort of 26 TRAP2 mice (13 males + 13 females) featured three experimental subgroups: food-deprived mice injected with 4-OHT upon refeeding (*n* = 9), food-deprived mice injected with vehicle upon refeeding (to control for leaky Cre activity, *n* = 9), and food-deprived mice injected with 4-OHT without refeeding (to control for PSTN activity during fasting, *n* = 8). These mice were refed 8 h after 4-OHT administration. In this experiment, all mice were injected with the hM3Dq vector and the effect of CNO was tested according to a between-subject design. For the induction of Cre activity, all mice were transferred to a clean, new cage (without food), and 24 h later, 4-OHT (or vehicle) was injected immediately prior to the placement of chow pellets in the wire lid of food-deprived cages. Deprivation-induced body weight loss was confirmed and vigorous interactions of refed mice with the food hopper were noted, but consumption measures were not collected to avoid interfering with the cages during the time window of Cre activation. An additional cohort of 16 TRAP2 mice (7 males + 9 females) featured three experimental subgroups: food-deprived mice injected with 4-OHT (*n* = 6), food-deprived mice injected with vehicle (to control for leaky Cre activity, *n* = 5), and ad libitum fed mice injected with 4-OHT (to control for baseline PSTN activity, *n* = 5).

A cohort of 46 *Tac1-*Cre mice (22 males + 24 females) featured three experimental subgroups injected with either the hM3Dq (*n* = 16), hM4Di (*n* = 13), or mCherry (*n* = 15) vectors. The effect of CNO was tested according to a between-subjects design.

A cohort of 19 *Crh*-Cre mice (10 males + 9 females) were all injected with the hM3Dq vector and were treated with either CNO or vehicle for a between-subject analysis. The treatment assigned to each mouse remained the same across all assays.

A cohort of 19 C57BL/6 J mice (12 males + 7 females) featured three experimental subgroups that were co-injected with the hM3Dq and KORD vectors (1:1 premixed cocktail) in the PSTN and the retrograde Cre vector in the CeA (*n* = 6), the BNST (*n* = 7) or the PVT (*n* = 6) for pathway-specific manipulations. In this experiment, all mice were injected with chemogenetic ligands (CNO or SalB) and their respective vehicles for within-subject analysis (i.e., each mouse was tested four times). CNO was tested first, then SalB. In each case, the order of ligand and vehicle administration was counterbalanced between mice.

A cohort of 6 *Tac1-*Cre mice (1 male + 5 females) and 7 *Crh*-Cre mice (3 males + 4 females) were injected with the Con/Fon hM3Dq vector into the PSTN and retrograde Flpo vector in the CeA. All mice were injected with CNO and vehicle for within-subject analysis.

A cohort of 10 *Tac1-*Cre mice (6 males + 4 females) and 10 *Crh*-Cre mice (5 males + 5 females) were injected with the Con/Fon hM3Dq vector into the PSTN and retrograde Flpo vector in the BNST. All mice were injected with CNO and vehicle for within-subject analysis.

### Stereotaxic surgery, histology, and behavioral testing

Methodological details are provided as Supplementary information.

### Statistics

Raw data was processed in Microsoft Excel and statistical analysis was performed using GraphPad Prism software. Experiments evaluating between-subject effects of CNO in TRAP2 (including mCherry counts) and *Tac1*-Cre cohorts were analyzed using ordinary one-way ANOVAs, with an alpha of 0.05. Multiple comparisons were conducted with the Tukey’s test for TRAP2 mice and with the Dunnett’s test for *Tac1*-Cre mice (using the mCherry group as control condition). Experiments comparing the effect of CNO versus vehicle were analyzed using either unpaired (*Crh*-Cre mice) or paired (pathway-targeted and Con/Fon cohorts) two-tailed *t*-tests, with an alpha of 0.05. In each graph, individual values are plotted, bars show group averages, and error bars represent standard error of the mean.

## Results

### The PSTN refeeding ensemble delays the latency to initiate feeding

**Fig. 1:**
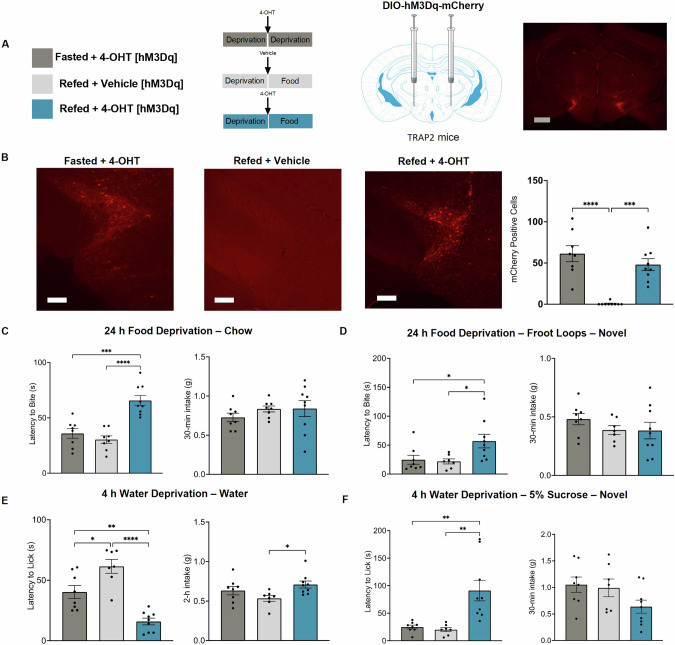
The ensemble of PSTN neurons activated by refeeding delays consumption of familiar and palatable foods but hastens water drinking. **A** Experimental design and representative image of mCherry fluorescence showing targeted recombination in the PSTN of a mouse injected with 4-OHT immediately prior to refeeding (scale bars: gray, 500 μm; white, 200 μm). **B** The number of PSTN mCherry-positive cells illustrates the extent of recombination across experimental conditions. Behavioral testing was performed 30 min after injection of the chemogenetic actuator CNO, and 24 h after food deprivation for chow (**C**) and Froot Loops (**D**), or 4 h after water deprivation for water (**E**) and sucrose (**F**). In each panel, the latency to initiate feeding/drinking is shown on the left and the amount of food/fluid consumed is shown on the right. Bars represent mean ± s.e.m. and individual values are overlaid. Data were analyzed using one-way ANOVA followed by Tukey’s *posthoc* comparisons when appropriate, *p < 0.05; **p < 0.01; ***p < 0.001, ****p < 0.0001.

Subpopulations of PSTN neurons become highly active in response to refeeding after food deprivation, as indexed by c-Fos induction [10, 13, 14]. To determine the functional significance of this neuronal ensemble, TRAP2 mice were injected with a Cre-dependent hM3Dq-encoding virus in the PSTN and administered 4-OHT upon refeeding following 24 h of food deprivation. In addition to this experimental group, a first control group was injected with 4-OHT in a fasted state to control for PSTN activity related to hunger, and another control group was injected with vehicle upon refeeding to control for leaky (i.e., 4-OHT-independent) Cre recombination (Fig. 1A). Native fluorescence of the mCherry reporter was used to estimate the size of the PSTN neuronal ensemble targeted in each of the three experimental groups. As expected, there were very few mCherry labeled cells in vehicle-injected mice (Fig. 1B). Accordingly, there was a significant main effect of group (F_2,23_ = 22.52, *p* < 0.0001), whereby mice injected with 4-OHT at the time of refeeding (*p* = 0.0001) and those injected with 4-OHT in a fasted state (*p* < 0.0001) had significantly more mCherry positive cells than vehicle-injected controls. The ensemble captured in a fasted state was not significantly different than that captured upon refeeding (*p* = 0.3691), providing a sizeable population of PSTN cells to inform on the functional specificity of the PSTN refeeding ensemble upon chemogenetic manipulation.

Mice were again deprived of food for 24 h and injected with CNO 30 min prior to regaining access to chow. There was a significant main effect of group on latency (F_2,22_ = 21.61, *p* < 0.0001) but not consumption (F_2,22_ = 0.75, *p* = 0.4859) (Fig. 1C). Re-activating the refeeding ensemble resulted in significantly longer latencies compared to both fasted controls (*p* = 0.0001) and vehicle controls (*p* < 0.0001).

When provided with novel Froot Loops after 24 h of food deprivation, there was again a significant main effect of group on latency (F_2,21_ = 4.82, *p* = 0.0189) but not consumption (F_2,21_ = 0.94, *p* = 0.4080) (Fig. 1D). Activation of the refeeding ensemble resulted in significantly longer latencies compared to both fasted controls (*p* = 0.0443) and vehicle controls (*p* = 0.0336).

We next sought to determine the impact of the PSTN refeeding ensemble on fluid consumption. Mice were deprived of water for 4 h at the onset of the dark phase and injected with CNO 30 min prior to regaining access to water. There was a significant main effect of group on latency (F_2,21_ = 24.86, *p* < 0.0001) and 2-h consumption (F_2,21_ = 3.48, *p* = 0.0497) (Fig. 1E). Interestingly, this is the only situation in which activation of a PSTN subpopulation resulted in a significant decrease in latency compared to vehicle controls. Activation of the refeeding ensemble resulted in significantly shorter latencies compared to vehicle controls (*p* < 0.0001) and to fasted controls (p = 0.0023), which also had significantly shorter latencies compared to vehicle controls (*p* = 0.0124). Activation of the refeeding ensemble ultimately led to significantly greater consumption of water compared to vehicle controls (*p* = 0.0393). This unique effect may reflect prandial thirst, which is normally triggered during food ingestion to anticipate changes in extracellular fluid osmolality following the absorption of solutes into the bloodstream [4].

When provided with a novel 5% sucrose solution after 4 h of water deprivation, there was a significant main effect of group on latency (F_2,21_ = 10.49, *p* = 0.0007) but not overall consumption (F_2,21_ = 2.61, *p* = 0.0970) (Fig. 1F). Activation of the refeeding ensemble resulted in significantly longer latencies compared to fasted controls (*p* = 0.0026) and vehicle controls (*p* = 0.0019).

We conducted a complementary experiment to corroborate these observations and include mice injected with 4-OHT in a sated state as additional control condition (Supplementary Fig. 1, details of statistical analysis are provided in Supplementary Information). In this cohort, mice injected with 4-OHT at the time of refeeding and those injected with 4-OHT in a sated state had significantly more mCherry positive cells than vehicle-injected controls, but the sated ensemble was significantly smaller than the refeeding ensemble (Supplementary Fig. 1B). Chemogenetic activation of the PSTN refeeding ensemble had similar effects as in the former cohort, with significantly longer latencies to eat familiar chow (Supplementary Fig. 1C) and novel Froot Loops (Supplementary Fig. 1D), shorter latency to drink water (Supplementary Fig. 1E), longer latency to drink novel sucrose (Supplementary Fig. 1F), and no difference in the amount of food or fluid consumed (Supplementary Fig. 1C–F) compared to vehicle controls. Furthermore, there was no significant difference between the latencies and intakes of sated controls vs. vehicle controls.

Taken together, these data demonstrate that the PSTN refeeding ensemble is functionally different from both the fasted and sated PSTN ensembles and drastically delays feeding initiation in hungry mice but does not control the amount of food consumed. This conclusion also applies to sucrose ingestion in thirsty mice. Furthermore, while both the PSTN refeeding and fasting ensembles accelerate water drinking, the refeeding ensemble exerts a far more drastic influence. It is possible that the refeeding, fasted, and sated ensembles partially overlap (i.e., a given PSTN cell may participate in two or three of these ensembles), but the refeeding ensemble stands out in its unique capacity to influence behavior. This functional specificity is not related to a threshold number of PSTN cells that need to be activated, as the fasted and refeeding ensembles had similar sizes. We next sought to examine which subpopulation(s) of PSTN neurons might be driving these striking latency effects.

### PSTN^*Tac1*^ neurons suppress both the initiation and the execution of consummatory behaviors

The PSTN contains two non-overlapping populations of neurons expressing either *Tac1* or *Crh*, and both populations are activated by refeeding [14]. PSTN^*Tac1*^ neurons were previously demonstrated to lower the amount of sucrose consumed by thirsty mice under conditions of novelty (neophobia) or sickness [12]. They also contribute to the reduction in meal frequency induced by anorexigenic hormones and reduce food intake upon chemogenetic or optogenetic stimulation [14]. In contrast, PSTN^*Crh*^ neurons do not exert such influence [14]. These studies, however, did not examine how manipulating the two populations might alter the delay to engage in consummatory behavior. We used Cre-dependent expression of chemogenetic actuators in the PSTN of *Tac1*-Cre and *Crh*-Cre mice to address this question.

**Fig. 2:**
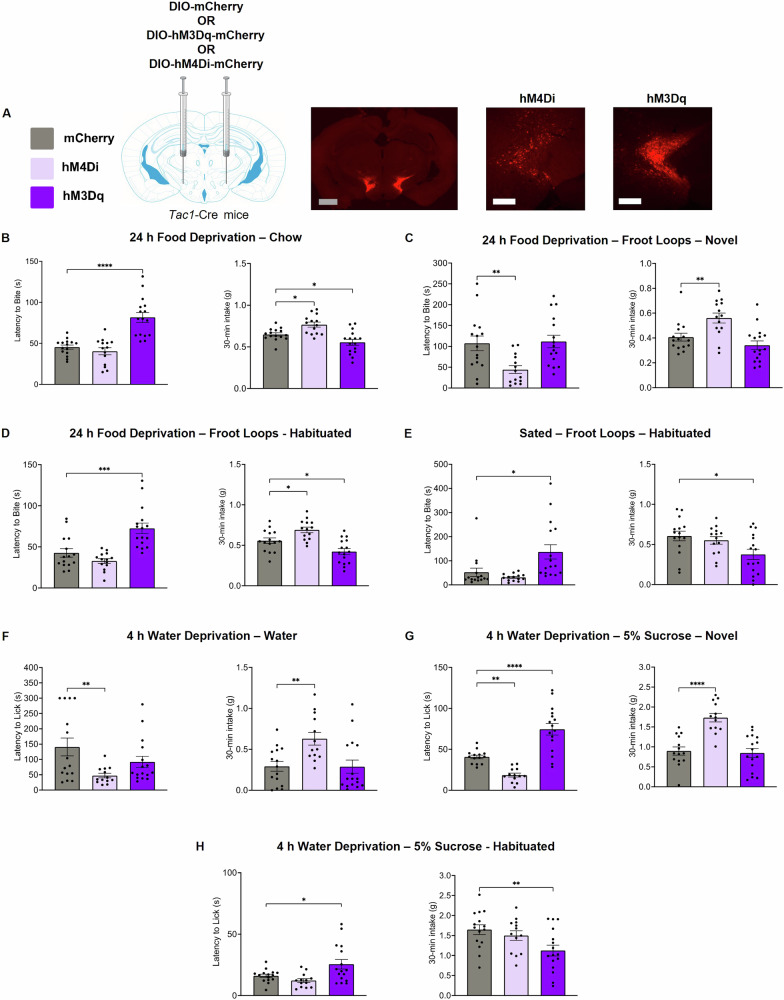
PSTN^*Tac1*^ neurons delay and reduce food and fluid consumption independently of caloric content, metabolic state, and novelty. **A** Experimental design and representative images of mCherry fluorescence in the PSTN illustrating expression of hM3Dq in PSTN^*Tac1*^ neurons (scale bars: gray, 500 μm; white, 200 μm). Behavioral testing was performed 30 min after injection of the chemogenetic actuator CNO, and 24 h after food deprivation for chow (**B**) and Froot Loops (**C**, **D**, no food deprivation but same circadian timepoint in **E**), or 4 h after water deprivation for water (**F**) and sucrose (**G**, **H**). Testing was performed upon first-time access to Froot Loops and sucrose (**C,** **G**), and again following habituation (**D**, **H**). Froot Loops consumption was also tested after habituation to scheduled access with concurrent ad libitum access to chow (**E**). In each panel, the latency to initiate feeding/drinking is shown on the left and the amount of food/fluid consumed is shown on the right. Bars represent mean ± s.e.m. and individual values are overlaid. Data were analyzed using one-way ANOVA followed by Dunnett’s *posthoc* comparisons to mCherry controls, *p < 0.05; **p < 0.01; ***p < 0.001; ****p < 0.0001.

Cre-dependent constructs encoding hM4Di, hM3Dq, or mCherry alone were virally transferred into the PSTN of *Tac1*-Cre mice (Fig. 2A). Mice were first assessed for refeeding with familiar chow following 24 h of food deprivation. There was a significant main effect of vector on both measures (latency: F_2,42_ = 24.85, *p* < 0.0001; consumption: F_2,42_ = 13.39, *p* < 0.0001) (Fig. 2B). Activation of PSTN^*Tac1*^ neurons significantly increased the latency to first bite of chow (*p* < 0.0001), while their inhibition had no significant effect (*p* = 0.66). PSTN^*Tac1*^ activation significantly reduced the amount of chow consumed (*p* = 0.0434). In contrast, PSTN^*Tac1*^ inhibition significantly increased chow consumption (*p* = 0.0138). These data indicate that chemogenetic stimulation of PSTN^*Tac1*^ neurons in food-deprived mice is sufficient to delay and reduce chow consumption despite the motivational state produced by hunger. They also show that the endogenous activity of PSTN^*Tac1*^ neurons during refeeding suppresses the consumption of familiar chow, thereby opposing the homeostatic drive to feed.

In a similar experimental design, mice were provided with a novel and presumably palatable high-sugar food, Froot Loops, after 24 h of food deprivation to assess the sensitivity of PSTN^*Tac1*^ neurons to other dimensions of consumption behaviors. There was a significant main effect of vector on both measures (latency: F_2,42_ = 6.46, *p* = 0.0036; consumption: F_2,42_ = 9.85, *p* = 0.0003) (Fig. 2C). Activation did not affect the latency to first bite of Froot Loops (*p* = 0.9670), nor the amount of Froot Loops consumed over 30 min (*p* = 0.3267). In contrast, inhibition resulted in significantly shorter latencies (*p* = 0.0086) and led mice to consume significantly more Froot Loops than controls (*p* = 0.0082). Interestingly, the results here demonstrate that novel palatable foods likely increase the endogenous activity of PSTN^*Tac1*^ neurons to its maximal extent given that no difference was observed in response to chemogenetic activation. This idea is bolstered by the ability of inhibition to reduce latencies to first bite to levels similar to familiar chow and to produce overconsumption compared to controls.

To disentangle the features of Froot Loops being simultaneously a novel and palatable food, mice were given both chow and Froot Loops ad libitum for 5 days to dampen novelty. Preference measures for chow versus Froot Loops (no CNO) were collected over a 24-h period during this habituation period and all mice demonstrated highly significant preference for Froot Loops compared to chow (*p* < 0.0001), thereby confirming the strong palatability of Froot Loops for these mice (Supplementary Fig. 2A). After habituation, mice were again tested with Froot Loops after 24 h of food deprivation. There was a significant main effect of vector on both measures (latency: F_2,42_ = 16.18, *p* < 0.0001; consumption: F_2,42_ = 14.17, *p* < 0.0001) (Fig. 2D). Activation of PSTN^*Tac1*^ neurons significantly increased latencies (*p* = 0.0003) and reduced intake (*p* = 0.0192) compared to controls. Inhibition did not significantly affect latency (*p* = 0.3238) but led mice to consume significantly more Froot Loops than controls (*p* = 0.0219). Strikingly, manipulations of PSTN^*Tac1*^ neurons while refeeding with habituated Froot Loops yielded effects similar to those observed for familiar chow. In both cases, activation caused a major hesitancy to begin eating and ultimately led to significantly less overall consumption. Inhibition, in contrast, did not increase the speed at which mice took their first bite but did produce overconsumption after 30 min. These data also strengthen the idea that PSTN^*Tac1*^ neurons may promote hyponeophagia via increased endogenous activity. Altogether, in hungry mice given access to food, the endogenous activity of PSTN^*Tac1*^ neurons delays feeding initiation selectively under conditions of novelty while it lowers the amount of food consumed regardless of novelty.

In the above-described experiments, food deprivation was used to motivate the mice to readily engage in food consumption. To control for the hunger state as a potential factor underlying our results, consummatory behaviors for habituated Froot Loops were also measured in a sated state, i.e., with ad libitum access to chow. Mice were habituated to receiving scheduled access to Froot Loops for 7–8 days to encourage consumption on test day. Upon CNO administration, there was a significant main effect of vector on both latency (F_2,42_ = 7.16, *p* = 0.0022) and consumption (F_2,42_ = 4.47, *p* = 0.0174) (Fig. 2E). Similar to hungry mice given access to familiar food, activation led sated mice to increase their latencies (*p* = 0.0122), while inhibition had no significant effect (*p* = 0.7091). Furthermore, activation significantly decreased consumption (*p* = 0.0128), while no difference was observed in response to inhibition (*p* = 0.7441). This outcome is compelling given the drastic difference in metabolic state of mice deprived of food for 24 h versus those in a sated state. These parallel results highlight that a state of hunger is not necessary for PSTN^*Tac1*^ neurons to drive feeding suppression, in line with data published by Kim et al. [14].

We next sought to determine the impact of chemogenetic manipulations of PSTN^*Tac1*^ neurons on fluid consumption. For these assays, mice were deprived of water for 4 h starting at the onset of the dark phase. When access to water was resumed after CNO administration, there was a significant main effect of vector on both measures (latency: F_2,41_ = 4.65, *p* = 0.0151; consumption: F_2,41_ = 6.621, *p* = 0.0032) (Fig. 2F). Activation did not affect latency to first lick (*p* = 0.1791) nor the amount of water consumed (*p* = 0.9990). Inhibition, however, resulted in significantly shorter latencies (*p* = 0.0078) and significantly higher consumption compared to controls (*p* = 0.0058). These observations reveal that PSTN^*Tac1*^ neurons exert potent inhibitory control over water consumption despite water restriction. Strikingly, the effect of inhibiting PSTN^*Tac1*^ neurons on the latency to rehydrate is comparable to the effect of activating the PSTN refeeding ensemble, which highlights a functional disconnect between these two PSTN subpopulations. This mismatch suggests that only a subset of PSTN^*Tac1*^ neurons may participate in the PSTN refeeding ensemble, in line with the *Fos*/*Tac1* colocalization analysis conducted by Kim et al. [14], and that PSTN^*Tac1*^ neurons do not belong to the ensemble driving prandial thirst.

To gain insight into whether PSTN^*Tac1*^ neurons are similarly sensitive to a novel and palatable fluid, mice were provided with a 5% sucrose solution after 4 h of water deprivation. There was a significant main effect of vector on both measures (latency: F_2,40_ = 34.58, *p* < 0.0001; consumption: F_2,40_ = 20.04, *p* < 0.0001) (Fig. 2G). Activation led to significantly longer latencies when compared to controls, similar to the effect seen with chow and habituated Froot Loops (*p* < 0.0001). In contrast, inhibition resulted in significantly shorter latencies, similar to the effect seen with novel Froot Loops (*p* = 0.0058). After 30 min, activation did not alter consumption (*p* = 0.9219), while inhibition led mice to consume significantly more sucrose solution compared to controls (*p* < 0.0001). The results here demonstrate that PSTN^*Tac1*^ neurons similarly modulate the consumption of both palatable solid and liquid foods.

As was done for Froot Loops, mice were habituated to the sucrose solution ad libitum for 5 days alongside a separate water bottle to disentangle novelty from the palatable nature of sucrose. Preference measures for water versus 5% sucrose (no CNO) were collected over a 72-h period and as expected, all mice demonstrated highly significant preference for sucrose compared to water (*p* < 0.0001) (Supplementary Fig. 2B). Upon CNO administration, there was a significant main effect of vector on both measures (latency: F_2,41_ = 6.36, *p* = 0.0039; consumption: F_2,41_ = 4.787, *p* = 0.0135) (Fig. 2H). Parallel to observations with habituated Froot Loops, activation significantly increased latencies (*p* = 0.0292), while no difference was observed with inhibition (*p* = 0.5328). Similarly, activation led mice to consume significantly less habituated sucrose (*p* = 0.0088), while inhibition had no significant effect (*p* = 0.6415). Building on the body of evidence collected in previous appetitive assays, inhibition of PSTN^*Tac1*^ neurons has a stronger overconsumption effect on novel palatable substances than when these items are habituated over time, likely indicating higher endogenous activity of PSTN^*Tac1*^ neurons during hyponeophagia.

### PSTN^*Crh*^ neurons promote the consumption of novel palatable substances

**Fig. 3:**
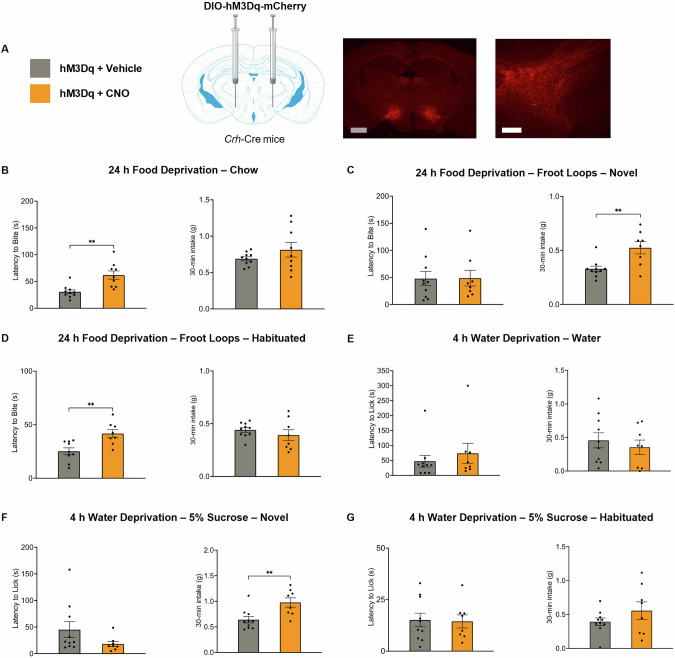
PSTN^*Crh*^ neurons delay refeeding of familiar foods and increase consumption of novel palatable substances. **A** Experimental design and representative images of mCherry fluorescence illustrating expression of hM3Dq in PSTN^*Crh*^ neurons (scale bars: gray, 500 μm; white, 200 μm). Behavioral testing was performed 30 min after injection of the chemogenetic actuator CNO or vehicle, and 24 h after food deprivation for chow (**B**) and Froot Loops (**C**, **D**), or 4 h after water deprivation for water (**E**) and sucrose (**F**, **G**). Testing was performed upon first-time access to Froot Loops and sucrose (**C**, **F**) and again following habituation (**D**, **G**). In each panel, the latency to initiate feeding/drinking is shown on the left and the amount of food/fluid consumed is shown on the right. Bars represent mean ± s.e.m. and individual values are overlaid. Data were analyzed using unpaired *t* tests, *p < 0.05; **p < 0.01.

In the PSTN, *Tac1*- and *Crh*-expressing neurons represent non-overlapping populations of the PSTN [14]. Accordingly, we tested whether PSTN^*Crh*^ neurons might exert a differential influence on consummatory behaviors compared to the PSTN^*Tac1*^ population. A Cre-dependent construct encoding the excitatory designer receptor hM3Dq was virally transferred into the PSTN of *Crh*-Cre mice (Fig. 3A).

Mice were deprived of food for 24 h and injected with CNO or vehicle 30 min prior to the beginning of experimental sessions. Mice were first assessed for refeeding with familiar chow (Fig. 3B). Activation of PSTN^*Crh*^ neurons by CNO resulted in significantly longer latencies compared to vehicle (t_18_ = 3.15, *p* = 0.0055). The amount of chow consumed in 30 min, however, was indistinguishable between treatments (t_18_ = 0.12, *p* = 0.9101). The results demonstrate that PSTN^*Crh*^ neurons can drive hesitancy to first bite of chow, but do not influence overall consumption.

Mice were provided with novel Froot Loops after 24 h of food deprivation to assess the influence of PSTN^*Crh*^ neurons on other dimensions of appetitive behaviors. Activation of PSTN^*Crh*^ neurons did not affect the latency to first bite (t_16_ = 0.05, *p* = 0.9627) but significantly increased Froot Loops consumption (t_16_ = 3.39, *p* = 0.0037) (Fig. 3C). These results demonstrate that activation PSTN^*Crh*^ neurons leads to overconsumption of a novel, palatable food, i.e., similar to the effect of inhibiting PSTN^*Tac1*^ neurons. Altogether, these observations suggest that the two populations oppose each other’s function in situations of hyponeophagia.

To disentangle the novelty of Froot Loops from their palatability, mice were given both chow and Froot Loops ad libitum for 5 days. After habituation, mice were again tested for Froot Loops after 24 h of food deprivation. As was observed with chow, activation resulted in significantly longer latencies (t_15_ = 3.37, *p* = 0.0042), with no significant effect on consumption (t_16_ = 0.95, *p* = 0.3574) (Fig. 3D).

We next sought to determine the impact of chemogenetic excitation of PSTN^*Crh*^ neurons on liquid consumption after 4 h of water deprivation. When access to water was resumed after CNO or vehicle administration, there was no significant effect of treatment on latency (t_16_ = 0.72, *p* = 0.4850) or consumption (t_16_ = 0.65, *p* = 0.5231) (Fig. 3E).

When provided with a novel, palatable 5% sucrose solution, there was no significant effect of treatment on latency (t_16_ = 1.54, *p* = 0.1432) but CNO increased sucrose consumption compared to vehicle (t_16_ = 3.15, *p* = 0.0062) (Fig. 3F). These results are congruent with the first-time Froot Loops assay, such that excitation of PSTN^*Crh*^ neurons significantly increase the consumption of novel, palatable solid and liquid substances without affecting the motivation to begin consuming.

Mice were habituated to the sucrose solution ad libitum for 5 days to disentangle novelty from the palatable nature of sucrose. There was no significant main effect of treatment on latency (t_16_ = 0.15, *p* = 0.8853) or consumption (t_16_ = 1.26, *p* = 0.2276) (Fig. 3G). Altogether, activation of PSTN^*Crh*^ neurons did not affect the consumption of familiar chow, habituated Froot Loops, or habituated sucrose and selectively increased first-time consumption of Froot Loops and sucrose. These results underscore the sufficiency of PSTN^*Crh*^ neurons to promote the consumption of novel palatable substances without altering the latency to initiate their ingestion.

### PSTN neurons projecting to the CeA suppress feeding initiation

**Fig. 4:**
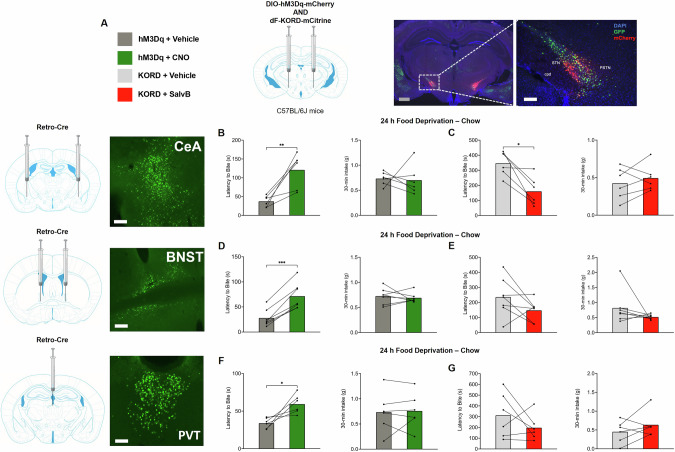
CeA- but not BNST- nor PVT-projecting PSTN neurons mediate refeeding delay. **A** Experimental design and representative images of GFP fluorescence illustrating Cre expression and mCherry fluorescence illustrating hM3Dq expression in the PSTN (scale bars: gray, 500 μm; white, 200 μm). Cre was expressed from a retrograde vector injected either in the CeA (**B**, **C**), BNST (**D**, **E**), or PVT (**F**, **G**). Behavioral testing was performed 30 min after injection of the chemogenetic actuators CNO (**B**, **D**, **F**) or SalB (**C**, **E**, **G**), or their respective vehicles, 24 h after food deprivation. Chow was presented either in the home cage (**B**, **D**, **F**) or in a novel arena (**C**, **E**, **G**). In each panel, the latency to initiate feeding/drinking is shown on the left and the amount of food/fluid consumed is shown on the right. Bars represent mean ± s.e.m. and individual values are overlaid. Data were analyzed using paired *t* tests, *p < 0.05; **p < 0.01; ***p < 0.001.

A dual-vector, pathway-specific chemogenetic approach was employed to determine the contribution of PSTN projection neurons. Previous neuroanatomic tracing demonstrated prominent projections from the PSTN to the CeA, BNST, and PVT [7, 14, 15], three structures that are involved in feeding regulation [30–33]. While PSTN^*Tac1*^ neurons send inputs to all three areas, food consumption is reduced by photoactivation of CeA-projecting and PVT-projecting, but not BNST-projecting, PSTN^*Tac1*^ neurons, highlighting the differential role of these pathways [14]. Furthermore, stimulation of PVT-projecting PSTN^VGluT2^ neurons reduces food intake [17]. We therefore sought to assess the influence of CeA-, BNST-, and PVT-projecting PSTN neurons in the control of feeding initiation. In C57BL/6 J mice, a retrograde Cre-expressing virus was injected into the CeA, BNST or PVT permitting the Cre-dependent expression of hM3Dq and KORD viral constructs co-injected into the PSTN to excite or inhibit those projections neurons upon administration of CNO or SalB, respectively (Fig. 4A).

Mice were deprived of food for 24 h and injected with chemogenetic ligands (CNO, SalB, or their respective vehicles) 30 min prior to refeeding with familiar chow. SalB testing was conducted in an open arena to increase feeding latency and facilitate the detection of downward shifts. PSTN^CeA^ activation significantly increased latency (t_5_ = 4.33, *p* = 0.0075) but did not alter consumption (t_5_ = 0.19, *p* = 0.8530) (Fig. 4B). Conversely, PSTN^CeA^ inhibition significantly shortened latency (t_5_ = 3.97, *p* = 0.0106) with no effect on consumption (t_5_ = 0.90, *p* = 0.4118) (Fig. 4C). PSTN^BNST^ activation also increased latency (t_6_ = 8.22, *p* = 0.0002) without altering consumption (t_6_ = 0.50, *p* = 0.6313) (Fig. 4D). PSTN^BNST^ inhibition had no significant effect on either latency (t_6_ = 1.71, *p* = 0.1365) or consumption (t_6_ = 1.27, *p* = 0.2496) (Fig. 4E). PSTN^PVT^ activation similarly increased latency (t_5_ = 3.35, *p* = 0.0202) but did not alter consumption (t_5_ = 0.23, *p* = 0.8241) (Fig. 4F). PSTN^PVT^ inhibition had no significant effect on either latency (t_5_ = 1.2, *p* = 0.2838) or consumption (t_5_ = 1.27, *p* = 0.2597) (Fig. 4G). Altogether, these data point to a prominent role of the endogenous activity of PSTN^CeA^ neurons in delaying food consumption initiation in hungry mice.

### CeA-projecting PSTN^*Tac1*^ neurons delay refeeding

**Fig. 5:**
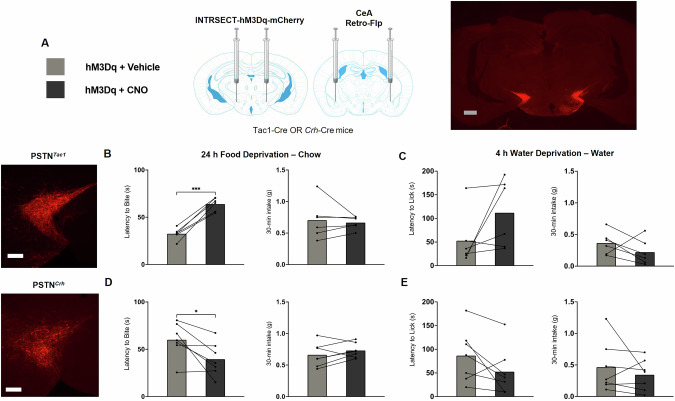
CeA-projecting PSTN^*Tac1*^ neurons delay refeeding while CeA-projecting PSTN^*Crh*^ neurons hasten refeeding. **A** Experimental design and representative images of mCherry fluorescence illustrating hM3Dq expression in CeA-projecting PSTN^*Tac1*^ (**B**, **C**) and PSTN^*Crh*^ (**D**, **E**) neurons (scale bars: gray, 500 μm; white, 200 μm). Behavioral testing was performed 30 min after injection of CNO or vehicle, and 24 h after food deprivation (**B**, **D**) or 4 h after water deprivation (**C**, **E**). In each panel, the latency to initiate feeding/drinking is shown on the left and the amount of food/fluid consumed is shown on the right. Bars represent mean ± s.e.m. and individual values are overlaid. Data were analyzed using paired *t* tests, *p < 0.05; ***p < 0.001.

Given that both PSTN^*Tac1*^ and PSTN^*Crh*^ neurons can delay feeding initiation in food-deprived mice given access to chow (Figs. 2B and 3B) and that they both project to the CeA [12, 14], either cell type may contribute to the influence of the PSTN^CeA^ projection on refeeding latency. To explore this possibility, we used a dual-vector, intersectional strategy to drive the expression of hM3Dq in PSTN^*Tac1*^ and PSTN^*Crh*^ neurons projecting to the CeA. This selective targeting was driven by the expression of Cre in *Tac1*-Cre or *Crh*-Cre mice respectively, the injection of a retrograde Flp-encoding vector in the CeA, and the injection of an INTRSECT (INTronic Recombinase Sites Enabling Combinatorial Targeting [29]) vector encoding hM3Dq-mCherry upon Cre AND Flp recombination in the PSTN (Fig. 5A).

Following 24-h food deprivation, activating CeA-projecting PSTN^*Tac1*^ neurons significantly increased the latency to initiate familiar chow consumption (t_5_ = 9.32, *p* = 0.0002) but did not affect the amount consumed (t_5_ = 0.434, *p* = 0.6822) (Fig. 5B). These results further underscore the importance of this molecularly defined projection given that activation of PSTN^*Tac1*^ neurons (Fig. 2B) and CeA-projecting neurons (Fig. 4B), in isolation or combined, significantly increases latency to first bite of familiar chow. Total consumption of chow was not affected by the activation of CeA-projecting PSTN^*Tac1*^ neurons, in contrast to the significant decrease observed with general PSTN^*Tac1*^ activation (Fig. 2B), but consistent with the outcome of activating CeA projectors as a whole (Fig. 4B). Most relevant to our goal of characterizing the neuronal subpopulations activated by food access in hungry mice, this pattern is identical to the effect of re-activating the PSTN refeeding ensemble (Fig. 1C and Supplementary Fig. 1C).

We then determined the effect of CeA-projecting PSTN^*Tac1*^ activation on water drinking. When access to water was resumed after CNO or vehicle administration, there was no significant effect of treatment on latency (t_5_ = 1.82, *p* = 0.1270) or consumption of water (t_5_ = 1.32, *p* = 0.2431) (Fig. 5C), indicating that these neurons do not contribute to the PSTN subpopulation triggering prandial thirst.

Activating CeA-projecting PSTN^*Crh*^ neurons after 24-h food deprivation significantly reduced the latency to initiate familiar chow consumption (t_6_ = 2.94, *p* = 0.0258) without affecting the amount consumed (t_6_ = 1.308, *p* = 0.2388) (Fig. 5D). CeA-projecting PSTN^*Crh*^ thereby stands out as the only population in our study sufficient to accelerate refeeding, which underscores not only an opposing role of *Tac1* and *Crh* subsets within the PSTN^CeA^ projection, but also the unique influence of CeA-projecting *Crh* cells among all PSTN^*Crh*^ neurons (Fig. 3B).

Activating CeA-projecting PSTN^*Crh*^ neurons after 4 h of water deprivation had no significant effect on the latency to initiate drinking (t_6_ = 1.94, *p* = 0.1011) or the amount of water consumed (t_6_ = 0.914, *p* = 0.3959) (Fig. 5E), similar to the results observed with general PSTN^*Crh*^ activation (Fig. 3E).

### BNST-projecting PSTN^*Crh*^ neurons hasten water drinking

**Fig. 6:**
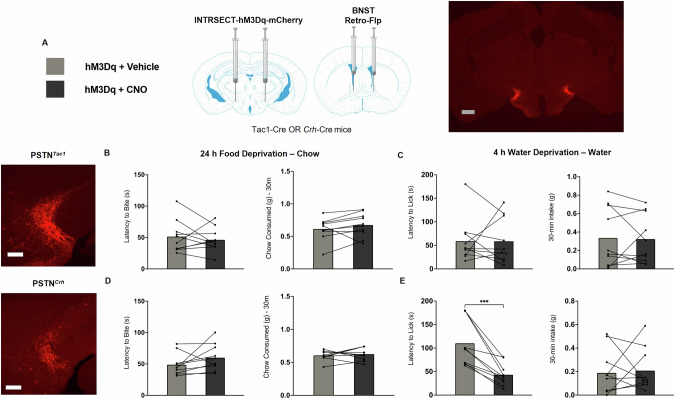
BNST-projecting PSTN^*Crh*^ neurons hasten water drinking. **A** Experimental design and representative images of mCherry fluorescence illustrating hM3Dq expression in BNST-projecting PSTN^*Tac1*^ (**B**, **C**) and PSTN^*Crh*^ (**D**, **E**) neurons (scale bars: gray, 500 μm; white, 200 μm). Behavioral testing was performed 30 min after injection of CNO or vehicle, and 24 h after food deprivation (**B**, **D**) or 4 h after water deprivation (**C**, **E**). In each panel, the latency to initiate feeding/drinking is shown on the left and the amount of food/fluid consumed is shown on the right. Bars represent mean ± s.e.m. and individual values are overlaid. Data were analyzed using paired *t* tests ***p < 0.001.

Given that both PSTN^*Tac1*^ and PSTN^*Crh*^ neurons project to the BNST [14] and that general activation of the PSTN^BNST^ pathway robustly delays feeding initiation in food-deprived mice given access to chow (Fig. 4D), we used the same dual-vector, intersectional strategy to drive the expression of hM3Dq in PSTN^*Tac1*^ and PSTN^*Crh*^ neurons projecting to the BNST and tease apart their respective roles on feeding and drinking behavior (Fig. 6A).

Following 24-h food deprivation, activating BNST-projecting PSTN^*Tac1*^ did not impact the latency to initiate familiar chow consumption (t_9_ = 0.66, *p* = 0.5247) or the amount consumed (t_9_ = 1.46, *p* = 0.1775) (Fig. 6B). These results demonstrate that BNST-projecting PSTN^*Tac1*^ neurons do not contribute to the robust latency increase triggered by general PSTN^*Tac1*^ activation (Fig. 2B), unlike their CeA-projecting counterparts (Fig. 5B).

We then determined the effect of BNST-projecting PSTN^*Tac1*^ activation on water drinking. When access to water was resumed after CNO or vehicle administration, there was no significant effect of treatment on latency (t_9_ = 0.02, *p* = 0.9868) or consumption of water (t_9_ = 0.20, *p* = 0.8496) (Fig. 6C).

Likewise, activating BNST-projecting PSTN^*Crh*^ neurons after 24-h food deprivation did not alter the latency to initiate familiar chow consumption (t_9_ = 1.65, *p* = 0.1325) or the amount consumed (t_9_ = 0.51, *p* = 0.6218) (Fig. 6D). These results show that neither BNST-projecting PSTN^*Tac1*^ nor PSTN^*Crh*^ are sufficient to drive the increase in latency observed following general PSTN^BNST^ activation (Fig. 4D), suggesting a role for yet another subpopulation of BNST-projecting PSTN neurons.

In contrast, activating BNST-projecting PSTN^*Crh*^ neurons after 4 h of water deprivation significantly shortened the latency to initiate drinking (t_9_ = 5.17, *p* = 0.0006) without altering the amount of water consumed (t_9_ = 0.25, *p* = 0.8056) (Fig. 6E), similar to the pattern evoked by the PSTN refeeding ensemble (Fig. 1E and Supplementary Fig. 1E). These results support the contribution of BNST-projecting PSTN^*Crh*^ neurons to the PSTN subpopulation triggering prandial thirst, and further emphasize functional heterogeneity with PSTN cell types as this effect was not captured by activating PSTN^*Crh*^ neurons as a whole.

## Discussion

The results of this study demonstrate that the PSTN exerts a profound effect on the motivation to initiate feeding and drinking. The PSTN is known to become active in response to diverse anorexigenic signals including binge-like refeeding following food deprivation, sickness, nutrient-deficient diets, novelty, and homeostatic hormonal signaling [8–14]. Our study highlights that the functional significance of this activation is to suppress the initiation of food consumption, an influence that extends to palatable solids and calorie-containing liquids, with no impact on the amount of food consumed (Fig. 1). In parallel, this ensemble *promotes* the initiation of water intake. We further find that these refeeding-sensitive cells are phenotypically similar to subpopulations of PSTN^*Tac1*^ neurons (Fig. 2) but distinct from PSTN^*Crh*^ neurons activated as a whole (Fig. 3). Furthermore, we show that PSTN^CeA^ neurons activated by food access in a hungry state play a prominent role in suppressing feeding initiation (Fig. 4). Specifically activating CeA-projecting PSTN^*Tac1*^ neurons was sufficient to delay refeeding, while activating CeA-projecting PSTN^*Crh*^ neurons hastened refeeding, highlighting the functional heterogeneity of PSTN subpopulations within a given anatomical pathway (Fig. 5). In contrast, activating BNST-projecting PSTN^*Crh*^ neurons exclusively hastened water drinking without impacting the latency to feed (Fig. 6).

Compared to previous studies that have investigated the influence of PSTN cell types and projections on consummatory behaviors [12, 14, 17–20], these results are the first to demonstrate a role of PSTN neurons in controlling the latency to initiate feeding or drinking, and the unique effects we observed on water drinking initiation are entirely novel to the field. Our study also disentangled the roles of palatability and novelty in the influence of PSTN^*Tac1*^ neurons on reinforcer consumption, with similar effects seen with familiar chow vs. Froot Loops or sucrose following habituation to these novel, palatable substances.

Strikingly, several of our experiments detected significant changes in latencies to first bite or first lick in the absence of changes in the amount of food or fluid consumed. This disconnect may reflect diverging biological underpinnings for appetitive motivation vs. real-time calorie or osmolarity homeostasis, or it could be related to the limited temporal resolution of our assays, which may have failed to detect differences in front loading behaviors. The latter limitation may explain why stimulating the PSTN^PVT^ projection did not alter food intake in our study, while it did in a previous study that used real-time measurement of food consumption in *Tac1*-Cre mice [14]. Another possible explanation for this discrepancy is that the influence of the PSTN^PVT^ pathway on food intake may be limited to sweet foods [14, 17].

Altogether, our data suggests that CeA-projecting PSTN^*Tac1*^ neurons represent a major component of the PSTN ensemble that drives hesitancy in hungry animals given access to food. By enhancing our understanding of the brain mechanisms controlling feeding onset, independently of consumption per se, this finding may have therapeutic relevance for the treatment of eating disorders. Activating CeA-projecting PSTN^*Tac1*^ neurons could help alleviate the craving component of bulimia nervosa and binge-eating disorders, while inhibiting this circuit may loosen pathological self-control in restrictive anorexia nervosa – and neither of these manipulations would be expected to negatively affect homeostatic food consumption. Future studies will aim to elucidate the molecular signaling events underlying the suppression of feeding initiation by CeA-projecting PSTN^*Tac1*^ neurons to afford pharmacological access to this circuit.

While PSTN neurons activated upon refeeding of hungry animals with familiar chow did not impact the amount of food consumed, our results from chemogenetic inhibition show that the endogenous activity of PSTN^*Tac1*^ neurons exerts a strong influence on consumption when mice are given access to a novel palatable food or fluid, consistent with the effect reported by Barbier et al. in mice given access to sucrose for the first time [12]. We found that this influence is reduced by habituation, which is consistent with reduced c-Fos induction in the PSTN of rats habituated to sucrose consumption [12], and further blunted by removing the state of metabolic need triggered by food deprivation. As expected, reducing the endogenous tone of PSTN^*Tac1*^ activity by using a familiar food or fluid was necessary for chemogenetic stimulation to significantly reduce consumption, in line with the effect of optogenetic stimulation of PSTN^*Tac1*^ neurons in ad libitum fed mice [14]. Interestingly, the endogenous activity of PSTN^*Tac1*^ neurons also suppressed water drinking, indicating that the influence of this subpopulation on consummatory behavior is independent of caloric content. This observation contrasts starkly with the influence of the PSTN refeeding ensemble on water intake (discussed in more detail below), indicating that these two PSTN subpopulations only have a partial overlap.

Another important outcome of our study was the opposing influence of PSTN^*Tac1*^ vs. PSTN^*Crh*^ neurons on palatable food consumption, which was suppressed by the former but increased by the latter. While previous studies had already identified an important role of PSTN^*Tac1*^ neurons in feeding suppression [12, 14], less is known regarding the physiological role of PSTN^*Crh*^ neurons [7]. PSTN^*Crh*^ neurons are activated by refeeding [14] and *Crh* expression in the PSTN is upregulated by an anorexia-inducing valine-deficient diet [9], but they do not influence food consumption ([14] and results reported here). Aside from consummatory behaviors, PSTN^*Crh*^ neurons have been shown to mediate defensive responses to acute predator threats and to regulate REM sleep [34]. Our observation that their activation promotes the consumption of sweet food and liquids suggests an important role in the control of hedonic feeding. Future studies will be needed to determine if this influence may extend to the consumption of other rewarding substances (e.g., addictive drugs) or experiences (e.g., social interaction).

Our results also uncover an unexpected implication of the PSTN in water drinking initiation. Re-activating the PSTN refeeding ensemble accelerated the onset of water consumption, and this phenotype was replicated when specifically activating BNST-projecting PSTN^*Crh*^ neurons. These data raise the possibility that BNST-projecting PSTN^*Crh*^ neurons may be connected to circuits controlling prandial thirst, such as subfornical organ *Nos1* neurons or posterior pituitary-projecting, vasopressin-secreting neurons [4, 35–37]. Our finding aligns with the recent discovery of a pathway-specific influence of insular cortex layer 5 projection neurons on water intake, whereby the subpopulation projecting to the PSTN promotes water licking in thirsty mice [38]. Interestingly, the shortening of drinking latency driven by the PSTN refeeding ensemble was selective for water, as an opposite effect was observed when mice were given access to a sucrose solution. Since latency modulation occurs before the mice get to sample the fluid’s taste, the differential effects of the PSTN refeeding ensemble on water vs. sucrose drinking initiation probably involve olfactory cues that enable the mice to predict the composition of the available solution [39].

While our study sought to both highlight and disentangle the heterogenous make-up of the PSTN, it is important to note that other cell types beyond *Tac1* and *Crh* neurons are found within the PSTN and are likely to influence the feeding and drinking behaviors reported here. A previous study determined that ~70% of PSTN^*Tac1*^ neurons and ~80% of PSTN^*Crh*^ neurons express *Fos* following refeeding [14], which justified focusing on these two populations in the context of the present study, but it also identified *Fos* in PSTN cells that express neither *Tac1* nor *Crh* (their proportion was not quantified). For instance, PSTN^*Adcyap1*^ neurons only partially overlap with the *Tac1* (~40%) and *Crh* (~20%) populations and may contribute to the PSTN refeeding ensemble given their known influence on feeding behavior [18]. Likewise, we chose to probe projections to the CeA, BNST, and PVT based on the established role of these brain areas in feeding control [30–33], but future studies will be needed to explore the role of PSTN projections to cortical, midbrain, and brainstem structures [14, 15].

The intersectional strategy we used for pathway-specific manipulations does not preclude that behavioral effects might be driven by collateral projections to other brain targets. Consistent with this possibility, there was a partial overlap in the phenotypes induced by the global manipulation of PSTN efferents to the CeA, BNST, and PVT (Fig. 4). However, there was a stark dichotomy in the outcome of stimulating cell-type *and* pathway-specific subpopulations (Fig. 5 vs. Fig. 6), which demonstrates the functional independence of these different circuits and invalidates a potential role of collaterals. Furthermore, while we were able to visualize mCherry-immunolabeled fibers in the targeted projection area (i.e., where the retrograde vector was injected), such labeling was not detected in other brain regions.

Another element that should be considered for the interpretation of our data relates to the cell-type specificity of Cre activity in the different mouse lines. We confirmed that there is minimal Cre-driven recombination in TRAP2 mice in the absence of 4-OHT (Fig. 1 and Supplementary Fig. 1). However, the size of the ensembles captured upon 4-OHT injection in sated mice and fasted, non-refed mice was larger than anticipated based on published cFos+ cell counting studies [10, 13, 14]. This discrepancy may result from the time window of Cre activity being longer than that of cFos protein expression [21] and/or from higher baseline PSTN activity during the dark phase [8]. Importantly, despite their sizes, the PSTN ensembles captured in sated and fasted mice did not trigger the same behavioral phenotypes as in refed mice, further demonstrating the functional specificity of the latter ensemble.

The fidelity and penetrance of Cre activity in *Tac1*-Cre and *Crh*-Cre lines can be visualized by double in situ hybridization in the offspring of crosses with the Cre reporter line Ai14 [40], as reported by the Allen Mouse Brain Connectivity Atlas (https://connectivity.brain-map.org/transgenic/, [41]). Overall, tdTomato mRNA extensively co-localizes with *Tac1* and *Crh* in adult PSTN cells of *Tac1*-Cre;Ai14 mice (experiment 180301447, images 32–34, and experiment 267222532, images 29–30) and *Crh*-Cre;Ai14 mice (experiment 182530933, image 12), respectively. We would like to note that experiment 156348221 has high and uneven background in the tdTomato channel, which had previously led us to conclude about poor fidelity of Cre activity in *Crh*-Cre mice [7]. In *Tac1*-Cre;Ai14 brains, there are tdTomato+ cells in the subthalamic nucleus, which is generally considered devoid of *Tac1* expression [16], but the tdTomato signal does co-localize with faint *Tac1* signal, consistent with very low levels of Cre being sufficient to induce reporter expression. In *Crh*-Cre;Ai14 PSTN, the tdTomato signal is strikingly bimodal, as it is intense and filling in some cells vs. faint and punctate in other cells. Overall, when including the cells with low, punctate tdTomato signal, there is excellent overlap between *Crh* and tdTomato within the PSTN for both fidelity and penetrance. However, there are *Crh*-negative cells with strong tdTomato labeling outside of the PSTN cluster, which emphasizes the importance of viral vector transduction anatomical accuracy. As expected, the regional selectivity of reporter expression (PSTN vs. adjacent brain areas) was higher in intersectional strategies (Figs. 4–6) than in mice injected with a single vector in the PSTN (Figs. 1–3).

In conclusion, our study identifies a novel circuit that suppresses feeding initiation despite hunger, which emphasizes the relevance of the PSTN as a key brain site in the control of appetite and food rejection [19] and has important implications for our understanding of the neural mechanisms that may be disrupted in eating disorders. It also identifies a novel circuit that promotes water drinking initiation, implicating for the first time the PSTN in the brain network driving prandial thirst.

## Supplementary information

Supplementary information

## Data Availability

All data supporting the findings of this study are available within the article and its supplementary information file.

## References

[1] Sternson SM, Eiselt AK. Three pillars for the neural control of appetite. Annu Rev Physiol. 2017;79:401–23. 27912679 10.1146/annurev-physiol-021115-104948

[2] Schwartz MW, Morton GJ. Obesity: keeping hunger at bay. Nature. 2002;418:595–7. 12167841 10.1038/418595a

[3] Zimmerman CA, Leib DE, Knight ZA. Neural circuits underlying thirst and fluid homeostasis. Nat Rev Neurosci. 2017;18:459–69. 28638120 10.1038/nrn.2017.71 PMC5955721

[4] Gizowski C, Bourque CW. The neural basis of homeostatic and anticipatory thirst. Nat Rev Nephrol. 2018;14:11–25. 29129925 10.1038/nrneph.2017.149

[5] Berthoud HR, Munzberg H, Morrison CD. Blaming the brain for obesity: integration of hedonic and homeostatic mechanisms. Gastroenterology. 2017;152:1728–38. 28192106 10.1053/j.gastro.2016.12.050 PMC5406238

[6] Rossi MA, Stuber GD. Overlapping brain circuits for homeostatic and hedonic feeding. Cell Metab. 2018;27:42–56. 29107504 10.1016/j.cmet.2017.09.021 PMC5762260

[7] Shah T, Dunning JL, Contet C. At the heart of the interoception network: Influence of the parasubthalamic nucleus on autonomic functions and motivated behaviors. Neuropharmacology. 2022;204:108906. 34856204 10.1016/j.neuropharm.2021.108906 PMC8688299

[8] Comoli E, Ribeiro-Barbosa ER, Negrao N, Goto M, Canteras NS. Functional mapping of the prosencephalic systems involved in organizing predatory behavior in rats. Neuroscience. 2005;130:1055–67. 15653000 10.1016/j.neuroscience.2004.10.020

[9] Zhu X, Krasnow SM, Roth-Carter QR, Levasseur PR, Braun TP, Grossberg AJ, et al. Hypothalamic signaling in anorexia induced by indispensable amino acid deficiency. Am J Physiol Endocrinol Metab. 2012;303:E1446–58. 23047987 10.1152/ajpendo.00427.2012 PMC3532465

[10] Zseli G, Vida B, Martinez A, Lechan RM, Khan AM, Fekete C. Elucidation of the anatomy of a satiety network: Focus on connectivity of the parabrachial nucleus in the adult rat. J Comp Neurol. 2016;524:2803–27. 26918800 10.1002/cne.23992 PMC5322267

[11] Zseli G, Vida B, Szilvasy-Szabo A, Toth M, Lechan RM, Fekete C. Neuronal connections of the central amygdalar nucleus with refeeding-activated brain areas in rats. Brain Struct Funct. 2018;223:391–414. 28852859 10.1007/s00429-017-1501-4 PMC5773374

[12] Barbier M, Chometton S, Pautrat A, Miguet-Alfonsi C, Datiche F, Gascuel J, et al. A basal ganglia-like cortical-amygdalar-hypothalamic network mediates feeding behavior. Proc Natl Acad Sci USA. 2020;117:15967–76. 32571909 10.1073/pnas.2004914117 PMC7354999

[13] Chometton S, Pedron S, Peterschmitt Y, Van Waes V, Fellmann D, Risold PY. A premammillary lateral hypothalamic nuclear complex responds to hedonic but not aversive tastes in the male rat. Brain Struct Funct. 2016;221:2183–208. 25863939 10.1007/s00429-015-1038-3

[14] Kim JH, Kromm GH, Barnhill OK, Sperber J, Heuer LB, Loomis S, et al. A discrete parasubthalamic nucleus subpopulation plays a critical role in appetite suppression. Elife. 2022;11:e75470. 35507386 10.7554/eLife.75470 PMC9119672

[15] Goto M, Swanson LW. Axonal projections from the parasubthalamic nucleus. J Comp Neurol. 2004;469:581–607. 14755537 10.1002/cne.11036

[16] Wallen-Mackenzie A, Dumas S, Papathanou M, Martis Thiele MM, Vlcek B, Konig N, et al. Spatio-molecular domains identified in the mouse subthalamic nucleus and neighboring glutamatergic and GABAergic brain structures. Commun Biol. 2020;3:338. 32620779 10.1038/s42003-020-1028-8 PMC7334224

[17] Zhang X, van den Pol AN. Rapid binge-like eating and body weight gain driven by zona incerta GABA neuron activation. Science. 2017;356:853–9. 28546212 10.1126/science.aam7100 PMC6602535

[18] Nagashima T, Tohyama S, Mikami K, Nagase M, Morishima M, Kasai A, et al. Parabrachial-to-parasubthalamic nucleus pathway mediates fear-induced suppression of feeding in male mice. Nat Commun. 2022;13:7913. 36585411 10.1038/s41467-022-35634-2 PMC9803671

[19] Zhang T, Perkins MH, Chang H, Han W, de Araujo IE. An inter-organ neural circuit for appetite suppression. Cell. 2022;185:2478–94.e28. 35662413 10.1016/j.cell.2022.05.007 PMC9433108

[20] Sanchez MR, Wang Y, Cho TS, Schnapp WI, Schmit MB, Fang C, et al. Dissecting a disynaptic central amygdala-parasubthalamic nucleus neural circuit that mediates cholecystokinin-induced eating suppression. Mol Metab. 2022;58:101443. 35066159 10.1016/j.molmet.2022.101443 PMC8844644

[21] DeNardo LA, Liu CD, Allen WE, Adams EL, Friedmann D, Fu L, et al. Temporal evolution of cortical ensembles promoting remote memory retrieval. Nat Neurosci. 2019;22:460–9. 30692687 10.1038/s41593-018-0318-7 PMC6387639

[22] Taniguchi H, He M, Wu P, Kim S, Paik R, Sugino K, et al. A resource of Cre driver lines for genetic targeting of GABAergic neurons in cerebral cortex. Neuron. 2011;71:995–1013. 21943598 10.1016/j.neuron.2011.07.026 PMC3779648

[23] Harris JA, Hirokawa KE, Sorensen SA, Gu H, Mills M, Ng LL, et al. Anatomical characterization of Cre driver mice for neural circuit mapping and manipulation. Front Neural Circuits. 2014;8:76. 25071457 10.3389/fncir.2014.00076 PMC4091307

[24] Feil R, Wagner J, Metzger D, Chambon P. Regulation of Cre recombinase activity by mutated estrogen receptor ligand-binding domains. Biochem Biophys Res Commun. 1997;237:752–7. 9299439 10.1006/bbrc.1997.7124

[25] Armbruster BN, Li X, Pausch MH, Herlitze S, Roth BL. Evolving the lock to fit the key to create a family of G protein-coupled receptors potently activated by an inert ligand. Proc Natl Acad Sci USA. 2007;104:5163–8. 17360345 10.1073/pnas.0700293104 PMC1829280

[26] Krashes MJ, Koda S, Ye C, Rogan SC, Adams AC, Cusher DS, et al. Rapid, reversible activation of AgRP neurons drives feeding behavior in mice. J Clin Invest. 2011;121:1424–8. 21364278 10.1172/JCI46229 PMC3069789

[27] Vardy E, Robinson JE, Li C, Olsen RH, DiBerto JF, Giguere PM, et al. A new DREADD facilitates the multiplexed chemogenetic interrogation of behavior. Neuron. 2015;86:936–46. 25937170 10.1016/j.neuron.2015.03.065 PMC4441592

[28] Tervo DG, Hwang BY, Viswanathan S, Gaj T, Lavzin M, Ritola KD, et al. A designer AAV variant permits efficient retrograde access to projection neurons. Neuron. 2016;92:372–82. 27720486 10.1016/j.neuron.2016.09.021 PMC5872824

[29] Fenno LE, Mattis J, Ramakrishnan C, Hyun M, Lee SY, He M, et al. Targeting cells with single vectors using multiple-feature Boolean logic. Nat Methods. 2014;11:763–72. 24908100 10.1038/nmeth.2996 PMC4085277

[30] Ciccocioppo R, Fedeli A, Economidou D, Policani F, Weiss F, Massi M. The bed nucleus is a neuroanatomical substrate for the anorectic effect of corticotropin-releasing factor and for its reversal by nociceptin/orphanin FQ. J Neurosci. 2003;23:9445–51. 14561874 10.1523/JNEUROSCI.23-28-09445.2003 PMC3035815

[31] Cai H, Haubensak W, Anthony TE, Anderson DJ. Central amygdala PKC-delta(+) neurons mediate the influence of multiple anorexigenic signals. Nat Neurosci. 2014;17:1240–8. 25064852 10.1038/nn.3767 PMC4146747

[32] Jennings JH, Rizzi G, Stamatakis AM, Ung RL, Stuber GD. The inhibitory circuit architecture of the lateral hypothalamus orchestrates feeding. Science. 2013;341:1517–21. 24072922 10.1126/science.1241812 PMC4131546

[33] Millan EZ, Ong Z, McNally GP. Paraventricular thalamus: gateway to feeding, appetitive motivation, and drug addiction. Prog Brain Res. 2017;235:113–37. 29054285 10.1016/bs.pbr.2017.07.006

[34] Tseng YT, Zhao B, Chen S, Ye J, Liu J, Liang L, et al. The subthalamic corticotropin-releasing hormone neurons mediate adaptive REM-sleep responses to threat. Neuron. 2022;110:1223–39.e8. 35065715 10.1016/j.neuron.2021.12.033

[35] Zimmerman CA, Lin YC, Leib DE, Guo L, Huey EL, Daly GE, et al. Thirst neurons anticipate the homeostatic consequences of eating and drinking. Nature. 2016;537:680–4. 27487211 10.1038/nature18950 PMC5161740

[36] Mandelblat-Cerf Y, Kim A, Burgess CR, Subramanian S, Tannous BA, Lowell BB, et al. Bidirectional anticipation of future osmotic challenges by vasopressin neurons. Neuron. 2017;93:57–65. 27989461 10.1016/j.neuron.2016.11.021 PMC5215952

[37] Augustine V, Lee S, Oka Y. Neural control and modulation of thirst, sodium appetite, and hunger. Cell. 2020;180:25–32. 31923398 10.1016/j.cell.2019.11.040 PMC7406138

[38] Takemoto M, Kato S, Kobayashi K, Song WJ. Dissection of insular cortex layer 5 reveals two sublayers with opposing modulatory roles in appetitive drinking behavior. iScience. 2023;26:106985. 37378339 10.1016/j.isci.2023.106985 PMC10291511

[39] Glendinning JI, Maleh J, Ortiz G, Touzani K, Sclafani A. Olfaction contributes to the learned avidity for glucose relative to fructose in mice. Am J Physiol Regul Integr Comp Physiol. 2020;318:R901–R16. 32160005 10.1152/ajpregu.00340.2019

[40] Madisen L, Zwingman TA, Sunkin SM, Oh SW, Zariwala HA, Gu H, et al. A robust and high-throughput Cre reporting and characterization system for the whole mouse brain. Nat Neurosci. 2010;13:133–40. 20023653 10.1038/nn.2467 PMC2840225

[41] Oh SW, Harris JA, Ng L, Winslow B, Cain N, Mihalas S, et al. A mesoscale connectome of the mouse brain. Nature. 2014;508:207–14. 24695228 10.1038/nature13186 PMC5102064

