# Supplementary material for: The parasubthalamic nucleus refeeding ensemble delays feeding initiation and hastens water drinking
Source: Mol Psychiatry. 2024 Jul 4;30(1):37–49. doi: 10.1038/s41380-024-02653-y (PMC11649566; doi:10.1038/s41380-024-02653-y)

**Supplementary Methods**

**Stereotaxic surgery**

All mice were anesthetized with isoflurane and placed in a stereotaxic frame (David Kopf Instruments, model 940). A small hole was drilled in the skull (David Kopf Instruments, 1474) and a 75-200 nL volume of viral vector was injected using a microinjector pump (World Precision Instruments, UMP3T-2) with an attached 10-μL NanoFil syringe (World Precision Instruments) fitted with a 33-gauge NanoFil blunted tip (World Precision Instruments, NF33BL). The following coordinates were used (AP from bregma, ML from midline, DV from skull, in mm): PSTN (AP ‑2.3, ML ± 1.1, DV -5.2), CeA (AP ‑1.2, ML ± 2.8, DV -4.5), BNST (AP 0.2, ML ± 1.0, DV -4.3), PVT (AP -1.7, ML ± 0, DV -3.5). The vector was infused at a rate of 0.1 μL/min with the needle left in place for 5 minutes post injection to minimize backflow. The scalp was sutured using surgical thread. Mice were left undisturbed for at least three weeks post-injection prior to behavioral testing.

**Histology**

Mice were anesthetized with chloral hydrate and perfused with cold phosphate buffered saline (PBS) followed by 3.7% paraformaldehyde (PFA). Brains were dissected and immersion fixed in PFA for 2 hours at 4°C, cryoprotected in 30% sucrose in PBS at 4°C until brains sank, flash frozen in 2-methyl-butane chilled on a dry ice ethanol slurry and stored at ‑80°C. Coronal 35-µm thick brain sections were sliced with a cryostat (Leica CM1950), collected in five series spanning appropriate brain regions in PBS containing 0.01% sodium azide, and stored at 4°C. Sections were then washed for 10 minutes in PBS, plated on Superfrost plus glass slides (Fisher Scientific, 1255015), and air-dried. Coverslips were mounted using DAPI-containing Vectashield Hardset medium (Vector Laboratories, H1500). Images were captured using a Keyence BZ-X700 fluorescence microscope. In all DIO cohorts, native fluorescence signals were used to assess the accuracy of stereotaxic targeting and exclude mistargeted mice from behavioral datasets accordingly (sample sizes reported in the “Experimental cohorts” section include well-targeted mice only). In pathway-targeted mice, we were not able to image mCitrine native fluorescence due to overlapping signal from eGFP-Cre in the green channel but used the mCherry signal to assess the location of the KORD/hM3Dq cocktail infusion. In the Con/Fon cohort, we used mCherry immunolabeling to enhance the signal (chicken anti-mCherry antibody, Abcam, ab205402, RRID AB_2722769, 1:5,000, overnight incubation at 4°C; goat anti-chicken conjugated to Alexa Fluor 568, Life Technologies, RRID AB_2534098, 1:500, 2-h incubation at room temperature). Counts of left and right hemisphere mCherry-labeled cells in TRAP2 mice were obtained with the Cell Counter tool of ImageJ (NIH), averaged, and used to evaluate the size of the refeeding-activated PSTN ensemble.

**Behavioral testing**

All behavioral testing was conducted during the active (dark) phase of the circadian cycle, 3-6 hours after lights had turned off. For all assays, CNO, SalB, or their respective vehicle was administered 30 min prior to the introduction of food/fluid. Latency to first bite/lick was recorded as a measure of motivation to consume, and the amount of food/fluid consumed was measured over 30 min (except for water consumption in TRAP2 mice, which was measured over 2 hours). Investigators were blind to experimental group allocation during data collection.

**Testing with solid foods**

A single pre-weighed food pellet (familiar chow) or 7 pieces of Froot Loop cereal (novel or habituated) were introduced in the cage. The wire lid, water bottle, and filter top were then put back in place. Latency to first bite was collected with a stopwatch, with the experimenter carefully distinguishing between movement of the food, burying of the food, and actual biting of the food which was accompanied by mastication sounds. The chow pellet or Froot Loops were weighed again 30 minutes later to measure amount of food consumed.

For food deprivation, mice were transferred to a new, clean home cage without food and testing was started 24 hours later.

Preference testing for chow versus Froot Loops was performed in *Tac1*-Cre mice, in the absence of chemogenetic manipulations. *Ad libitum* fed mice were given two chow pellets and 20 pieces of Froot Loop cereal that were measured before and after a 24-hour period. 19 of 46 mice consumed all the Froot Loops during the testing period, the calculated preference values are therefore an underestimate of actual preference.

For Froot Loops habituation, mice were given chow and Froot Loops *ad libitum* for another 3 days after preference testing.

For experiments in sated *Tac1*-Cre mice, mice were given scheduled access to 3 pieces of Froot Loops 3 hours into the dark phase for 7 or 8 days prior to the experiment, along with *ad libitum* access to chow.

For SalB testing in pathway-targeted mice, mice were tested in an open arena (Taconic Transit Cage, 56-cm long × 40-cm wide × 18-cm deep) lined with 2 cm of fresh bedding, instead of their home cage. A single food pellet was secured onto a platform located in the center of the arena (see 28 for details). This novel environment was used to make the mice more hesitant to initiate feeding and facilitate the detection of a latency reduction.

**Testing with liquids**

Prior to liquid testing, mice were habituated to 50-mL conical tubes fitted with rubber stoppers and sippers for 7 days. On the morning of testing, water bottles in each cage were removed at the onset of the dark phase for 4 hours prior to testing. Mice consume very little water during their inactive phase and a significant portion of their daily water intake at the beginning of their active phase (29, 30), such that mice deprived of water for the first 4 hours after lights turn off have a high motivation to consume water when access is resumed. At the time of testing, a single, pre-weighed 50-mL conical tube containing normal drinking water or a 5% (w/v) solution of sucrose (Sigma-Aldrich 84097) was inserted in the wire lid, and the filter top was put back in place. Latency to first lick was collected with a stopwatch, with attention paid to differentiating whisking from licking behaviors. A 5-minute time limit was used for latency measurements. The bottle was weighed again 30 minutes (or 120 minutes for water testing in TRAP2 mice) later. A control cage with no mouse was used as spill control and the weight lost in that cage was subtracted from all other cages.

Preference testing for water versus sucrose was performed in *Tac1*-Cre mice, in the absence of chemogenetic manipulations. All mice were given two 50-mL conical tubes in their home cage for 72 hours, one containing water and one containing 5% sucrose solution. Bottles were weighed before and after the experiment. The 72-hour preference test was used to habituate mice to the sucrose solution for subsequent experiments.

**Supplementary Results**

TRAP2 mice were injected with a Cre-dependent hM3Dq-encoding virus in the PSTN and administered 4-OHT upon refeeding following 24 hours of food deprivation. In addition to this experimental group, a first control group was injected with 4-OHT in a sated state to control for baseline PSTN activity, and another control group was injected with vehicle upon refeeding to control for leaky (i.e., 4-OHT-independent) Cre recombination (**Supplementary Figure 1A**). Native fluorescence of the mCherry reporter was used to estimate the size of the PSTN neuronal ensemble targeted in each of the 3 experimental groups. As expected, there were very few mCherry labeled cells in vehicle-injected mice (**Supplementary Figure 1B**). Accordingly, there was a significant main effect of group (F_2,12_=14.68, p=0.0006), whereby mice injected with 4-OHT at the time of refeeding (p=0.0004) and those injected with 4-OHT in a sated state (p=0.0447) had significantly more mCherry positive cells than vehicle-injected controls. The ensemble captured in a sated state, which reflects baseline PSTN activity, was significantly smaller than that captured upon refeeding (p=0.0482) but provides a sizeable population of PSTN cells to inform on the functional specificity of the PSTN refeeding ensemble upon chemogenetic manipulation.

Mice were again deprived of food for 24 hours and injected with CNO 30 minutes prior to regaining access to chow. There was a significant main effect of group on both latency (F_2,13_=13.70, p=0.0006) and consumption (F_2,13_=4.04, p=0.0433) (**Supplementary Figure 1C**). Re-activating the refeeding ensemble resulted in significantly longer latencies compared to both sated controls (p=0.0009) and vehicle controls (p=0.0040). Consumption tended to be reduced compared to sated controls (p=0.0651) and vehicle controls (p=0.0841).

When provided with novel Froot Loops after 24 hours of food deprivation, there was a significant main effect of group on latency (F_2,13_=9.77, p=0.0026) but not consumption (F_2,13_=1.91, p=0.1871) (**Supplementary Figure 1D**). Activation of the refeeding ensemble resulted in significantly longer latencies compared to both sated controls (p=0.0066) and vehicle controls (p=0.0058).

We next sought to determine the impact of the PSTN refeeding ensemble on fluid consumption. Mice were deprived of water for 4 hours at the onset of the dark phase and injected with CNO 30 minutes prior to regaining access to water. There was a significant main effect of group on latency (F_2,12_=6.55, p=0.0119) but not 2-h consumption (F_2,12_=0.36, p=0.7046) (**Supplementary Figure 1E**). Interestingly, this is the only situation in which activation of a PSTN subpopulation resulted in a significant decrease in latency, here compared to vehicle controls (p=0.0119) and trending for sated controls (p=0.0778).

When provided with a novel 5% sucrose solution after 4 hours of water deprivation, there was again a significant main effect of group on latency (F_2,12_=6.82, p=0.0105) but not overall consumption (F_2,13_=0.08, p=0.9225) (**Supplementary Figure 1F**). Activation of the refeeding ensemble resulted in significantly longer latencies compared to sated controls (p=0.0240) and vehicle controls (p=0.0209).

**Supplementary Figure 1. The ensemble of PSTN neurons active in sated mice does not influence food and fluid consumption latency or amount.** **A.** Experimental design and representative image of mCherry fluorescence showing targeted recombination in the PSTN of a mouse injected with 4-OHT immediately prior to refeeding (scale bars: gray, 500 μm; white, 200 μm). **B.** The number of PSTN mCherry-positive cells illustrates the extent of recombination across experimental conditions. Behavioral testing was performed 30 minutes after injection of the chemogenetic actuator CNO, and 24 hours after food deprivation for chow (**C**) and Froot Loops (**D**), or 4 hours after water deprivation for water (**E**) and sucrose (**F**). In each panel, the latency to initiate feeding/drinking is shown on the left and the amount of food/fluid consumed is shown on the right. Bars represent mean ± s.e.m. and individual values are overlaid. Data were analyzed using one-way ANOVA followed by Tukey’s *posthoc* comparisons when appropriate, *, p<0.05; **, p<0.01; ***, p<0.001.


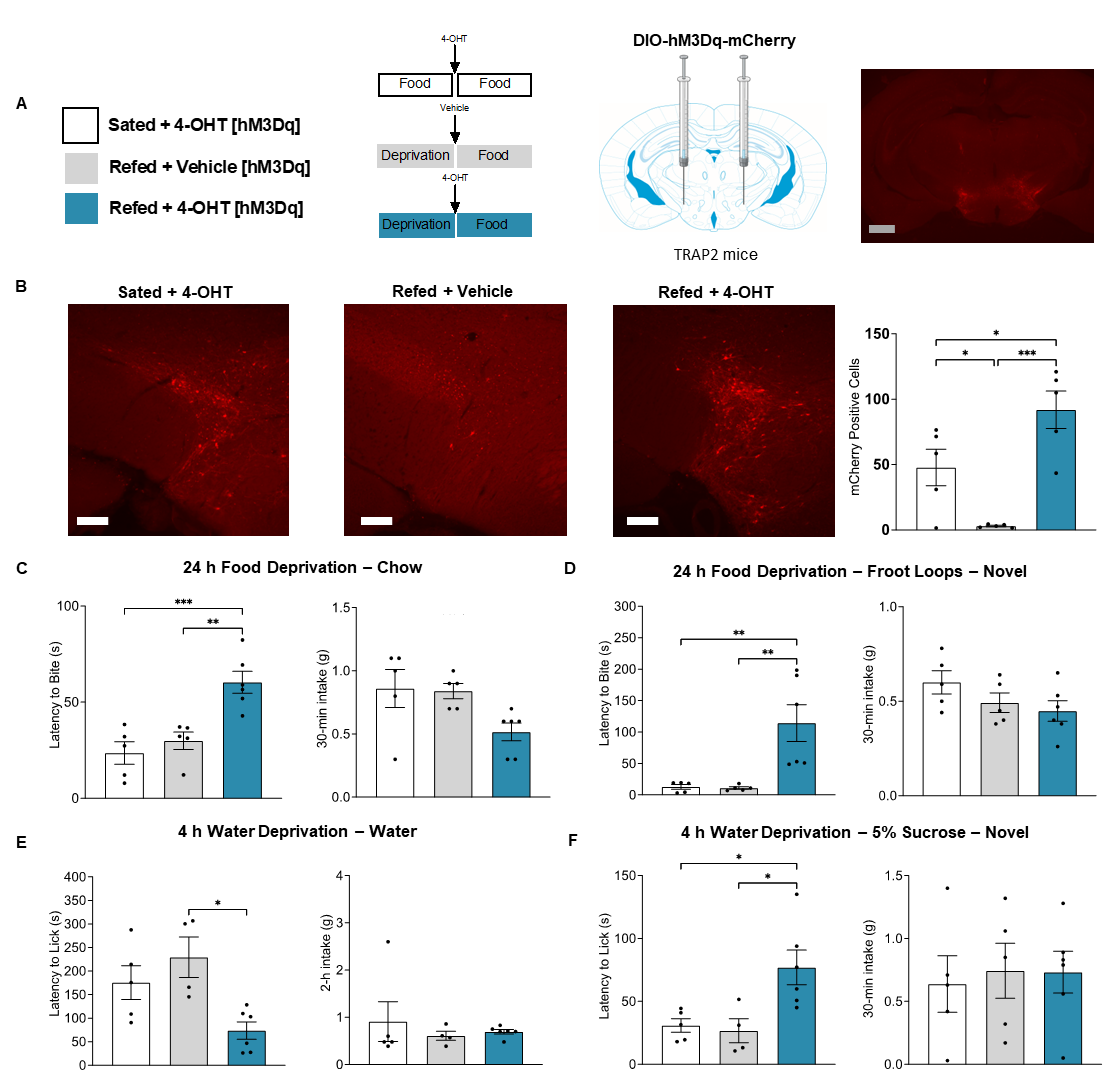


**Supplementary Figure 2.** **Mice demonstrate robust preference for Froot Loops and sucrose.** To interpret experimental data obtained upon chemogenetic manipulation, all mice were given the choice to consume (**A**) regular chow and Froot Loops during a 24-hour period, or (**B**) water and a 5% sucrose solution during a 72-hour period. No CNO was administered. Consumption is measured in grams on the left and average percent of total consumption is illustrated on the right. Data were analyzed using two-way ANOVA followed by Tukey’s *posthoc* comparisons, ****, p<0.0001.


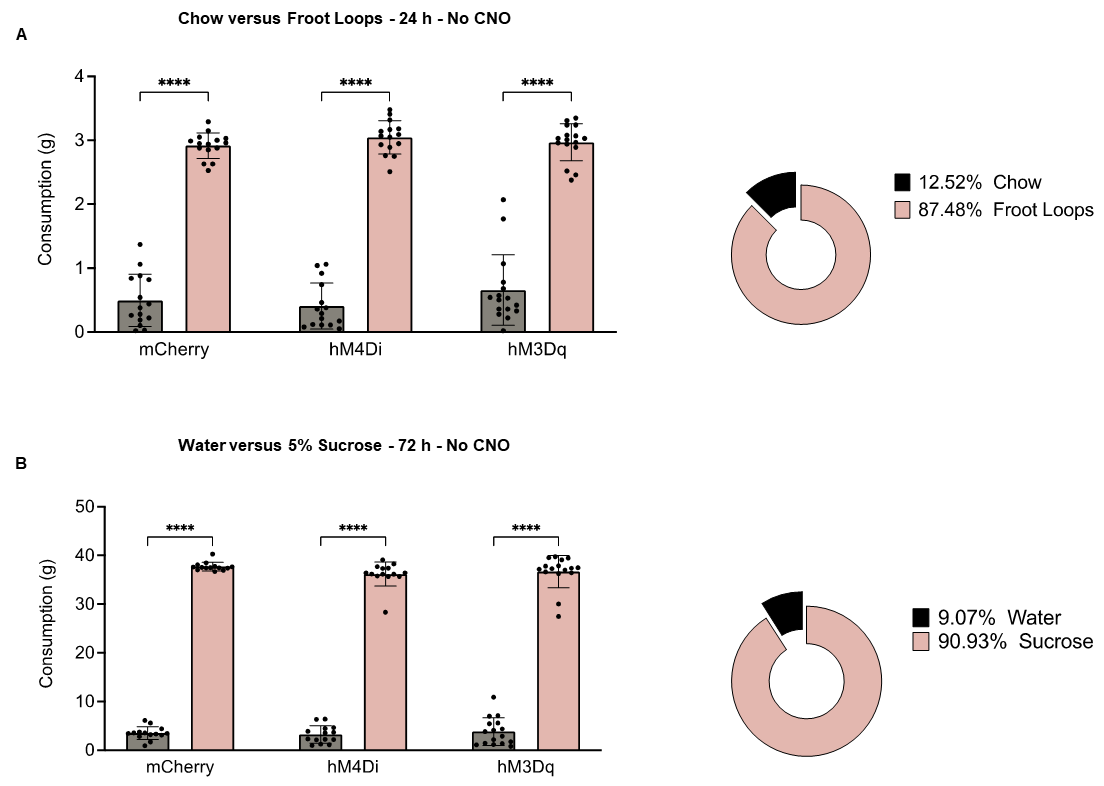

Supplement: Supplementary file 1 — Supplementary information [file 41380_2024_2653_MOESM1_ESM.docx]
